# Are the London Declaration’s 2020 goals sufficient to control Chagas disease?: Modeling scenarios for the Yucatan Peninsula

**DOI:** 10.1371/journal.pntd.0006337

**Published:** 2018-03-19

**Authors:** Bruce Y. Lee, Sarah M. Bartsch, Laura Skrip, Daniel L. Hertenstein, Cameron M. Avelis, Martial Ndeffo-Mbah, Carla Tilchin, Eric O. Dumonteil, Alison Galvani

**Affiliations:** 1 Public Health Computational and Operations Research, Johns Hopkins Bloomberg School of Public Health, Baltimore, MD, United States of America; 2 Global Obesity Prevention Center, Johns Hopkins Bloomberg School of Public Health, Baltimore, MD, United States of America; 3 Center for Infectious Disease Modeling and Analysis, Yale School of Public Health, New Haven, CT, United States of America; 4 Department of Tropical Medicine, School of Public Health and Tropical Medicine, Tulane University, New Orleans, LA, United States of America; Universidad de Buenos Aires, ARGENTINA

## Abstract

**Background:**

The 2020 Sustainable Development goals call for 100% certified interruption or control of the three main forms of Chagas disease transmission in Latin America. However, how much will achieving these goals to varying degrees control Chagas disease; what is the potential impact of missing these goals and if they are achieved, what may be left?

**Methods:**

We developed a compartmental simulation model that represents the triatomine, human host, and non-human host populations and vector-borne, congenital, and transfusional *T*. *cruzi* transmission between them in the domestic and peridomestic settings to evaluate the impact of limiting transmission in a 2,000 person virtual village in Yucatan, Mexico.

**Results:**

Interruption of domestic vectorial transmission had the largest impact on *T*. *cruzi* transmission and prevalence in all populations. Most of the gains were achieved within the first few years. Controlling vectorial transmission resulted in a 46.1–83.0% relative reduction in the number of new acute Chagas cases for a 50–100% interruption in domestic vector-host contact. Only controlling congenital transmission led to a 2.4–8.1% (30–100% interruption) relative reduction in the total number of new acute cases and reducing only transfusional transmission led to a 0.1–0.3% (30–100% reduction). Stopping all three forms of transmission resulted in 0.5 total transmission events over five years (compared to 5.0 with no interruption); interrupting all forms by 30% resulted in 3.4 events over five years per 2,000 persons.

**Conclusions:**

While reducing domestic vectorial, congenital, and transfusional transmission can successfully reduce transmission to humans (up to 82% in one year), achieving the 2020 goals would still result in 0.5 new acute cases per 2,000 over five years. Even if the goals are missed, major gains can be achieved within the first few years. Interrupting transmission should be combined with other efforts such as a vaccine or improved access to care, especially for the population of already infected individuals.

## Author summary

While World Health Organization’s (WHO) London Declaration on Neglected Tropical Diseases has proposed 2020 goals of 100% certified interruption or control of the three main forms of Chagas disease transmission (vectorial, congenital, and transfusional) in Latin America, the impact of achieving and/or missing these goals is not known. Policy makers need to know the potential impact of missing these goals on disease incidence and prevalence. If they are achieved, decision makers need to know what may be left to adequately inform policies and the future for controlling Chagas disease. Our compartmental simulation model suggests that achieving the 2020 goals would still result in 25 new acute cases per 100,000 over five years. However, substantial gains could still be garnered within one year by interrupting transmission to varying degrees, so the goals should still be pursued.

## Introduction

While the World Health Organization’s (WHO) London Declaration on Neglected Tropical Diseases has proposed 2020 goals of “100% of countries certified with no intradomiciliary transmission”, “100% of countries with certification of transfusional transmission interrupted”, and “100% of countries with control of congenital transmission” regarding the three main forms of Chagas disease transmission in Latin America[1], the question remains: what will be the impact of achieving these goals to varying degrees be on Chagas disease? Interruption of domestic transmission (often measured by infections in children under 5 years of age) is thought to play a key role in controlling Chagas disease (i.e., reduction in Chagas disease burden), which is caused by the protozoan parasite *Trypanosoma cruzi*.[2–5] While previous studies have tried to elucidate the mechanisms of transmission or evaluate particular interventions[6–14], none to our knowledge have specifically tried to evaluate the impact of achieving the 2020 goals. In fact, many existing studies preceded the formulation and announcement of the goals.

Moreover, not all locations may be able to achieve the 2020 goal, which does not necessarily mean aspiring to them is not worthwhile. Some regions have yet to implement policies or mandate control programs[1] (e.g., Mexico has no national control program[4]), while other regions have programs that are not consistent from year-to-year and region-to-region (e.g., geographic variations in control activities in Ecuador[15]). Additionally, low attendance to perinatal care can hinder adequate diagnosis and treatment[16] of pregnant women and infants, and compliance with universal screening of blood donors is not always 100%.[17] Furthermore, Chagas policies may be thwarted by decentralization (i.e., movement of authority from a central to a local government).[1, 18] Therefore, knowing the impact of partially achieving the goals to varying degrees would be helpful.

Really assessing the potential impact of achieving the 2020 goals would need a computational model that incorporates all the complexities. For example, a model would need to incorporate all the other relevant routes of transmission (e.g., vectorial, tranfusional, and congenital[2, 3, 19]) to help determine how much disease would persist if vectorial transmission were interrupted. It also should include various vector habitats (e.g., domestic, peridomestic, and sylvatic) to help determine the impact of reinfestation. The Yucatan in Mexico can serve a good sample location as Mexico has not received certification, this region has some of the highest levels of Chagas in the country, and its main vector has more than one habitat (i.e., domestic, peridomestic, and sylvatic) which allows us to capture re-infestation dynamics that may thwart the 2020 goals. Therefore, our team developed a dynamic model of *T*. *cruzi* transmission among vectors (*Triatoma dimidiata*) and human and non-human hosts in Yucatan, Mexico and evaluated different levels of achieving the three transmission related 2020 goals on Chagas disease prevalence and number of new acute human cases.

## Methods

### Model structure

**Fig 1:**
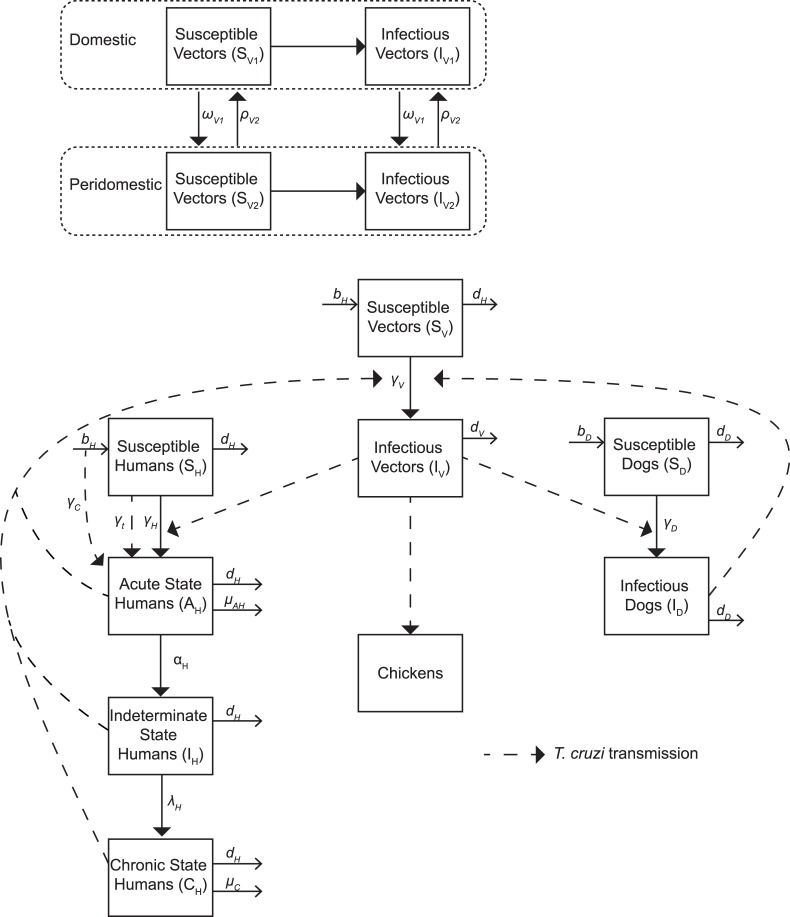
Model structure.

We developed a deterministic compartmental model (Fig 1) using Python (Python Software Foundation, Wilmington, DE) to represent vector and host populations involved in *T*. *cruzi* transmission and included triatomines, human hosts, non-human hosts (i.e., dogs), and dead-end hosts (i.e., chickens) to simulate vector-borne transmission between these populations in both domestic and peridomestic habitats, as well as congenital and transfusion/organ transplantation transmission. The S1 Text provides additional model details (including equations representing transitions between compartments). The model ran in monthly time steps (i.e., t = 1 month or 30 days) and was simulated across a 50-year period. During each time step, probabilities and rates determined the number of individuals in each compartment.

Triatomine bugs could be susceptible (not infected with *T*. *cruzi* and able to become infected) or infectious (infected with *T*. *cruzi* and able to transmit to vertebrate hosts upon biting). Upon feeding on an infectious host (human and viable non-human), a susceptible bug had a probability of becoming infected with *T*. *cruzi*, conditional on the disease state of the host. The number of triatomine bugs (*N*_*V*_) in the model was determined from the carrying capacity, or the number of bugs sustainable in each habitat. The number of susceptible triatomines entering the domestic or peridomestic population was dependent on the vector birth rate, carrying capacity, and number of triatomines in each habitat (S1 Text).

Each member of the human population (*N*_*H*_) could be in any of the following mutually exclusive disease states (Fig 1): susceptible (not infected with *T*. *cruzi* and able to become infected), acute Chagas disease (infected with *T*. *cruzi* and able to transmit, exhibiting mild and nonspecific symptoms, but in some cases can show specific symptoms such as Romaña’s sign or can be serious and life-threatening, and having microscopically detectable parasitemia for 6 to 8 weeks[19]), indeterminate Chagas disease (infected with *T*. *cruzi*, able to transmit, but showing no symptoms, i.e., asymptomatic), and symptomatic chronic Chagas disease (infected with *T*. *cruzi*, able to transmit, and showing symptoms of chronic disease such as cardiomyopathy and/or megaviscera). Upon a feeding contact by an infectious triatomine, a susceptible human had a probability of becoming infected with *T*. *cruzi* via contamination with bug feces during or immediately after the feeding. This is represented in the vector-borne force of infection (S1 Text). Based on the clinical progression of disease in humans[2, 19], all new infections start in the acute state. Pregnant women had a probability of transmitting Chagas to their infants upon birth, with newborns becoming infected based on the congenital force of infection (S1 Text). Additionally, a proportion of humans receiving a blood transfusion or organ transplant had a probability of becoming infected with *T*. *cruzi*, based on the transfusion force of infection (S1 Text). We assumed that once infectious, persons were considered always infectious in the absence of treatment. Those in the acute and symptomatic chronic states of disease had probabilities of Chagas-related mortality.

Dogs (*N*_*D*_) served as reservoir hosts for *T*. *cruzi* and could be either susceptible or infected, with a susceptible dog becoming infected upon the bite of an infected vector at a rate depending on the force of infection (S1 Text). Dogs were considered competent transmitters of *T*. *cruzi* (i.e., susceptible triatomines could become infected upon biting an infected dog). Chickens (*N*_*C*_) served as dead end hosts and could not transmit *T*. *cruzi* back to vectors, as they are unable to become infected with *T*. *cruzi*.[20]

Our model included transmission in both domestic and peridomestic habitats, which vary by vector-host contact rates, and allowed for the movement of triatomines between them (e.g., re-infestation). Vectorial transmission in our model was governed by the vectorial force of infection (S1 Text). Consistent with other models of vector-borne diseases[21], this is a function of: (1) the triatomine biting rate, (2) the triatomine feeding proportion for each host type in each habitat, (3) the probability of transmission from vector to susceptible host, (4) the probability of transmission from infected host to susceptible bug, (5) the proportion of hosts in each habitat, and (6) the number of hosts in each habitat. Transmission probabilities from vector to host varied with host species, while triatomine biting rates were assumed to be constant.

### Yucatan, Mexico

Despite having one of the greatest burdens of Chagas disease worldwide, Mexico has not yet undertaken a national vector control program[4] and only started mandatory serological screening in 2012[17]. In 2010, approximately an estimated 876,458 people were infected and 23.5 million were at risk for infection[22], with 88% of the population potentially exposed to at least one competent vector species[23]. These cases result in an estimated $32.3 billion in societal costs over their lifetime.[24] Yucatan State has one of the highest Chagas burdens in Mexico. Chagas is endemic throughout the peninsula, with 12–25 cases reported per 100,000 population over the last several years.[25, 26] Additionally, the Yucatan is home to only one main vector species, *Triatoma dimidiata*, which can be found in the domestic, peridomestic, and sylvatic environments, and typically infests houses on a seasonal basis with limited ability to colonize.[27, 28] Thus, the domestic and peridomestic transmission cycle are fueled by the sylvatic transmission of *T*. *cruzi* and house invasion by infected bugs. Currently, there are no vector intervention or control strategies in place in the Yucatan. Thus, this endemic setting, with no programs currently in place and home to a vector that can reinfest homes, is an ideal location to fully estimate the impact of the 2020 goal.

### Data sources

Our model was populated and calibrated to simulate *T*. *cruzi* transmission in a rural village (*N*_*H*_ = 2,000) in Yucatan, Mexico. Table 1 shows our key input parameter values and sources. The number of dogs (*N*_*D*_ = 617) was based on the ratio of dogs to humans[29, 30], while the number of chickens (*N*_*C*_ = 250) was based on the proportion of households with chickens and the number of persons per household[28]. The carrying capacity was set at 50 bugs per person (consistent with previous work[9]), yielding a *T*. *dimidiata* population size of 99,885. Our model was calibrated to assume a median *T*. *cruzi* prevalence value of 32.5% in *T*. *dimidiata*[27, 31–38], and seroprevalence estimates of 1.85% in humans[4, 38–47], and 14.58% in dogs[31, 41, 42, 48–51]. As transmission probabilities (i.e., from vectors to humans and dogs, and from dogs and humans to triatomines) and *T*. *dimidiata* feeding proportions across host species are highly variable and/or not well defined in the literature, these parameters were calibrated to available empirical data for the Yucatan (Table 1 and S1 Text).

**Table 1:** Key model input parameters, values, and sources.

Parameter	Symbol	Value	Source
**Probabilities (% per month)**			
Developing chronic Chagas disease given indeterminate phase (% over 20 years)		25	[19]
Proportion time spent in domestic settings for humans (%)	*f*_H_	90	Assumption
Proportion time spent in domestic settings for dogs (%)	*f*_D_	23	[30]
Transmission to dogs given bite of infected vector (% per bite)	ε	0.00271 to 0.00346	Calibrated††
Transmission to humans given bite of infected vector (% per bite)	ε_H_	0.000111 to 0.000125	Calibrated††
Transmission from acute stage to triatomine (% per bite)°	Θa	49.28 to 65.71	Calibrated††
Transmission from indeterminate/chronic stage to triatomine (% per bite)°	Θi	1.60 to 1.64	Calibrated††
Transmission from dog to triatomine (% per bite)°	Θd	19.000 to 19.016	Calibrated††
Triatomine feeding proportion for humans in domestic settings	p_HD_	86.6 to 95.0	Calibrated††
Triatomine feeding proportion for humans in peridomestic settings	p_HP_	43.1 to 50.0	Calibrated††
Ratio of triatomine feeding proportion for chickens:dogs in peridomestic settings‡	p_D_:p_C_	50.6:49.4 to 55.0:45.0	Calibrated††
Congenital transmission given birth from infected mother (% per birth)°	ε_*c*_	10.0 to 23.8	Calibrated††
Relative prevalence of women of reproductive age as compared to the general population	*φ_wr_*	80.0 to 107.0	Calibrated††
Transmission via infected blood transfusion or organ transplant°	ε_*t*_	8.0 to 21.4	Calibrated††
Human receiving a blood transfusion or organ donation	*p_t_*	0.00109 to 0.00983	Calibrated††
**Rates (per month)**			
Triatomine biting rate	β	6	[63]
Triatomine birth rate (eggs hatching per month)^	b_v_	32.13	[64]
Triatomine death rate*	d_v_	0.036959468	[64, 65]
Triatomine in peridomestic habitat moves to domestic habitat	ρ	0.0205936	[13]
Triatomine in domestic habitat moves to peridomestic habitat	ω	0.0205936	[13]
Human birth rate	b_H_	same as natural death rate and Chagas death rate	Assumption
Human death rate	d_H_	0.001111111	[66]
Chagas related mortality during the acute stage	μ_AH_	0.007178811	[67]
Movement from the acute phase to the indeterminate phase	*α*_H_	0.04688	[19, 67]
Movement from the indeterminate phase to chronic phase	λ_H_	0.001198675	[2, 19]
Rate of Chagas related mortality during chronic stage	μ_CH_	0.00680	[68]
Dog birth rate	b_D_	same as death rate	Assumption
Dog death rate	d_D_	0.013888889	[69]
**Numbers**			
Number of humans	N_H_	2000	Assumption
Number of dogs	N_D_	= N_H_/3.24	[29, 30]
Number of chickens†	N_C_	= (N_H_/4)*0.5	[28]
Carrying capacity		50 bugs per person	Assumption

††Calibrated value ranges represent the minimum and maximum values that meet the target baseline output values according to our calibration method (S1 Text). This range of input values was tested for every scenario to obtain a mean output value for each scenario.

°Calibrated values fall within range of limited published literature [17, 62, 70–72]

‡Calibrated values for remainder of feeding proportion after humans, where chicken proportion is higher than for dogs following the literature[61, 72]

^Assumes maximum number of eggs laid per day and egg hatching rate (90%) from literature[64] and assumes 7% of vector population are adult females

*Calculated using the adult life span and days from egg to adult to determine total life span

†Assumes 50% of households have chickens and that there are 4 to 5 persons per household

### Experimental scenarios

We evaluated the impact of interrupting vector-borne transmission in the domestic setting, and congenital and transfusional transmission to varying degrees to achieve the 2020 goals. We modeled each as an attenuation of the force of infection (S1 Text). For vector-borne transmission, we modeled this as a reduction in the contact rate between humans and triatomines only in the domestic setting; that is, we attenuated the force of infection by a specified amount and account for the proportion of transmission due to domestic vectors (S1 Text). As the goal is to evaluate the 2020 goals and not the way in which these are achieved, these reductions served as a proxy to represent a variety of ways transmission could be interrupted in the domestic settings (e.g., housing improvements, indoor residual spraying, bed nets) and for congenital transmission (e.g., screening and treatment). Sensitivity analyses evaluated the degree to which transmission was interrupted for the three types (0% to 100%). Additional sensitivity analyses further evaluated each calibrated parameter at their low and high calibrated values (Table 1). We also varied the movement of triatomines to and from the peridomestic and domestic settings (±50%), as this can vary with many factors.[13, 52] Model outcomes are the number of new/acute human cases (which reflects transmission) and the overall prevalence of human cases (which reflects the general disease burden).

## Results

### No transmission reduction

**Fig 2:**
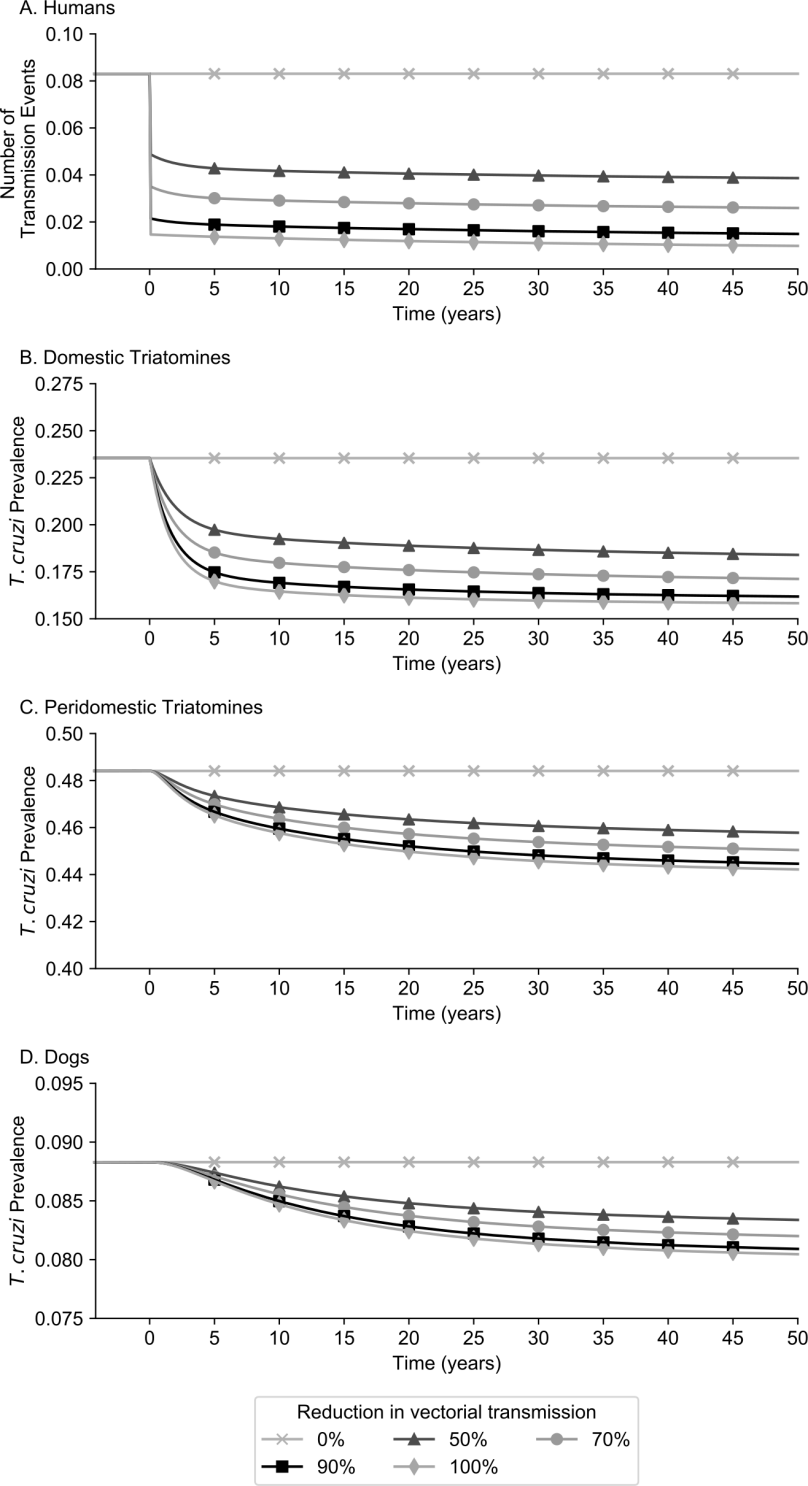
Impact of the degree of reduction of only vector-host contact rates with continuous interruption of domestic vectorial transmission on *T*. *cruzi* transmission events and seroprevalence over time in A) humans, B) domestic triatomines, C) peridomestic triatomines, and D) dogs.

**Fig 3:**
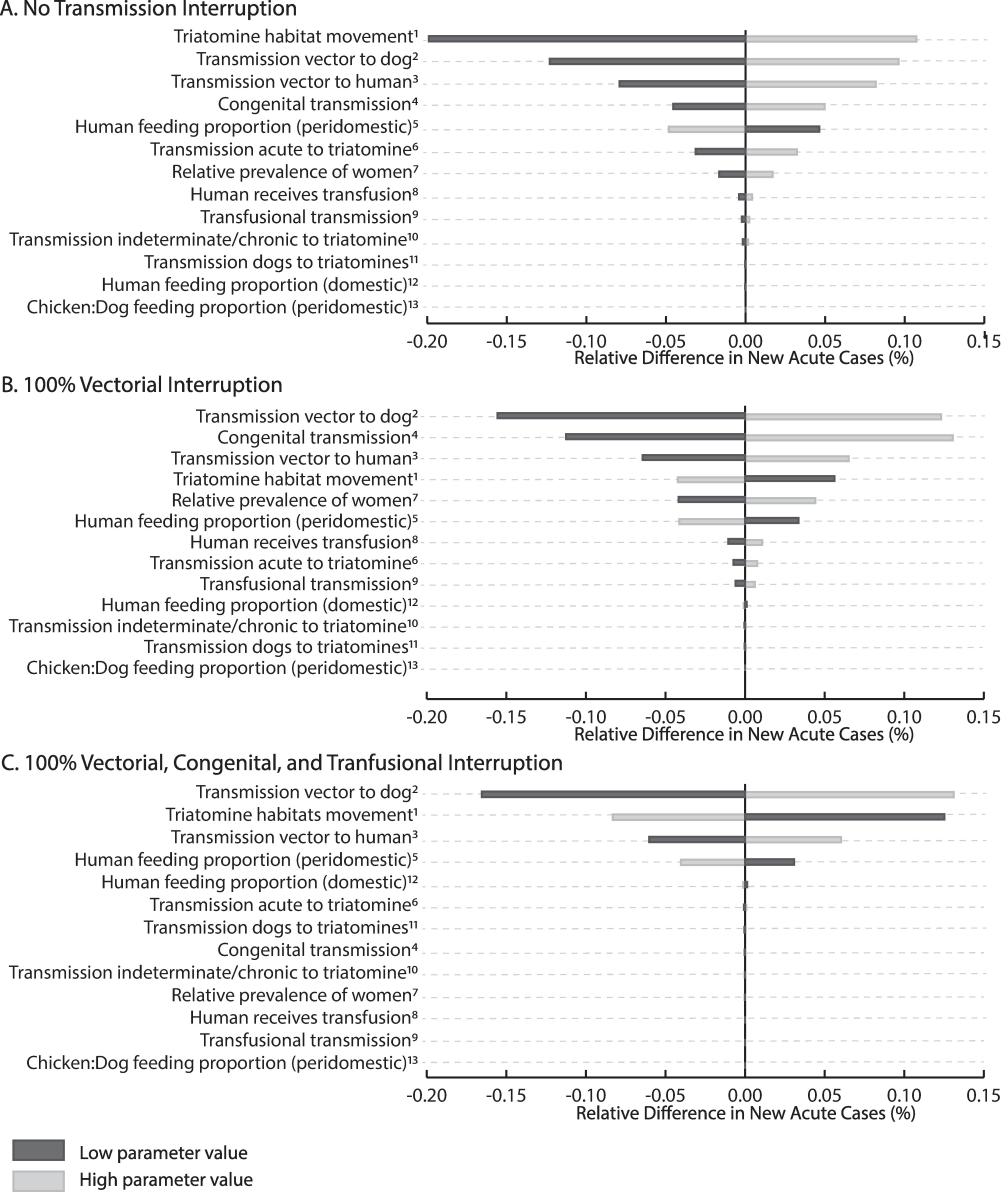
Impact of calibrated parameters on the number of new monthly acute cases (i.e., monthly transmission events) measures in percent relative difference for A) no transmission interruption; B) 100% interruption of vectorial transmission, and C) 100% interruption of vectorial, congenital, and transfusional transmission. The x-axis is the percent relative change from a base case in which all parameters on the y-axis are held at their middle value (0.080, 0.009, and 0.007, for panel A, B, and C, respectively). The width of the bar shows the range for the impact each had when varied from its minimum and maximum value. Numbers are the rank of each parameter with no transmission interruption to show how the rank changes with interruption. Note: month events are after new equilibrium is reached in the event of interruption.

With no interruption in any form of transmission, *T*. *cruzi* prevalence in humans remained stable at 1.8%, with 1.0 new acute case each year (i.e., transmission event), so that at any given point in time there were 1.5 acute cases, 30.6 indeterminate cases, and 4.6 chronic Chagas disease cases in the population of 2,000 persons (Table 2). *T*. *cruzi* prevalence in triatomines remained stable at 23.5% and 48.4% for those in the domestic and peridomestic habitat, respectively (Fig 2B and 2C), while the prevalence of *T*. *cruzi* in dogs remained stable at 8.8% (Fig 2D). Fig 3A shows the maginitude of impact of each of various parameters on the resulting number of new monthly acute infections. Movement of triatomines between habitats had the largest impact, resulting in 0.064 to 0.089 transmission events per month (for -50% to +50% of the baseline value); followed by transmission from vector to dog and vector to humans. Varying the three most impactful parameters (the transmission from vector to dog, transmission from vector to human, and the proportion at which triatomine feed on humans) to their extreme values resulted 0.72 to 1.18 new acute cases each year and a prevalence of 17.7% to 26.0% in domestic triatomines and of 35.9% to 52.6% in periodomestic triatomines.

**Table 2:** Impact of interruption of domestic vectorial transmission to varying degrees on the number of humans in each Chagas disease state at given time points over the 50-year period (all scenarios assume 0% reduction in congenital and transfusional transmission) in Yucatan, Mexico.

	Reduction in Only Domestic Vectorial Transmission				
	0%	50%	70%	90%	100%
Year 0					
Acute	1.51	1.51	1.51	1.51	1.51
Indeterminate	30.56	30.56	30.56	30.56	30.56
Chronic	4.63	4.63	4.63	4.63	4.63
Year 5					
Acute	1.51	0.82	0.6	0.39	0.3
Indeterminate	30.56	29.29	28.85	28.45	28.25
Chronic	4.63	4.6	4.59	4.58	4.57
Year 15					
Acute	1.51	0.75	0.52	0.32	0.23
Indeterminate	30.56	25.97	24.51	23.22	22.62
Chronic	4.63	4.32	4.22	4.12	4.08
Year 25					
Acute	1.51	0.73	0.5	0.3	0.21
Indeterminate	30.56	23.31	21.08	19.12	18.21
Chronic	4.63	3.93	3.72	3.52	3.44
Year 35					
Acute	1.51	0.72	0.49	0.29	0.19
Indeterminate	30.56	21.21	18.4	15.93	14.8
Chronic	4.63	3.57	3.25	2.96	2.83
Year 45					
Acute	1.51	0.71	0.48	0.28	0.18
Indeterminate	30.56	19.57	16.3	13.45	12.14
Chronic	4.63	3.26	2.85	2.49	2.32

### Varying the degree that vectorial transmission is reduced

Fig 2 and Table 2 show the impact of only domestic vectorial interruption to varying degrees over time on *T*. *cruzi* transmission events, prevalence, and the number of acute, indeterminate, and chronic Chagas disease cases. The largest reductions in prevalence were seen within the first year of reducing vector-host contact with the impact becoming stable by year five, regardless of the degree of reduction. Fig 2A shows the reduction in total *T*. *cruzi* transmission events in humans. Over the course of one year, a 50% to 100% reduction in domestic vector-host contact resulted in a 42.8% to 82.5% relative reduction in the number of new acute Chagas cases; this increased to a relative reduction of 46.1% to 83.0% over five years (Fig 2A and Table 2). Even with a sharp reduction in the total number of new transmission events, the number of Chagas disease cases remained relatively stable over time, with a decrease in the number of indeterminate and chronic disease cases taking approximately 12 years to manifest (Table 2). Fig 3B shows the impact of varying parameters on new acute cases with a 100% reduction in domestic vector-host. As shown, the rank order of parameters change from the no interruption scenario so that transmission from vector to dog, congenital transmission, and transmission from vector to humans had the largest impact. Varying transmission from vector to dog, transmission from vector to human, and the proportion at which triatomines feed on humans to their extremes resulted 0.08 to 0.13 new acute cases each year.

Among triatomines (Fig 2B and 2C), *T*. *cruzi* prevalence among domestic triatomines experienced relative decreases of 16.2%, 21.3%, 25.8%, and 27.7% compared to no reduction for vector-host reductions of 50%, 70%, 90%, and 100% respectively, after five years, while peridomestic triatomines garnered relative reductions up to 3.9%. After 50 years, the prevalence among triatomines ranged from 10.9% to 17.4% in the domestic (15.8% for base assumptions, Fig 2B) and from 30.4% to 48.6% in the peridomestic (44.2% for base assumptions, Fig 2C) settings under all conditions tested with a 100% vector-host reduction. The prevalence of *T*. *cruzi* among dogs decreased to 8.7% at 5 years when attenuating domestic transmission by 50% to 100% (Fig 2D).

The differences between scenarios in Fig 2 and Table 2 show that gains can be achieved by increasing the degree of vector-host interruption at different points in time. For example, increasing from 50% to 100% in year 3 would result in 2.0 total transmission events by year 5 compared to 2.7 events per 2,000 persons.

### Varying the degree that congenital transmission is reduced

After five years, only controlling congenital transmission led to a 2.4% (30% reduction) to 8.1% (100% reduction) relative reduction in the total number of new acute cases. This resulted in 0.1 to 0.4 fewer total transmission events, respectively. However, controlling only congenital transmission had very little impact on *T*. *cruzi* prevalence in triatomines and dogs, with maximum relative reductions of 0.9%, 0.2%, and 0.1% in domestic triatomine, peridomestic triatomine, and dog seroprevalences, respectively, after five years.

### Varying the degree that transfusional transmission is reduced

Reducing only transfusional transmission had minimal impact on the number of new acute cases and no impact on *T*. *cruzi* prevalence in any population. The relative reduction in the total number of new acute cases ranged from 0.1% to 0.3% (30% to 100% reduction) over five years.

### Varying the degree that all transmission routes are reduced

**Fig 4:**
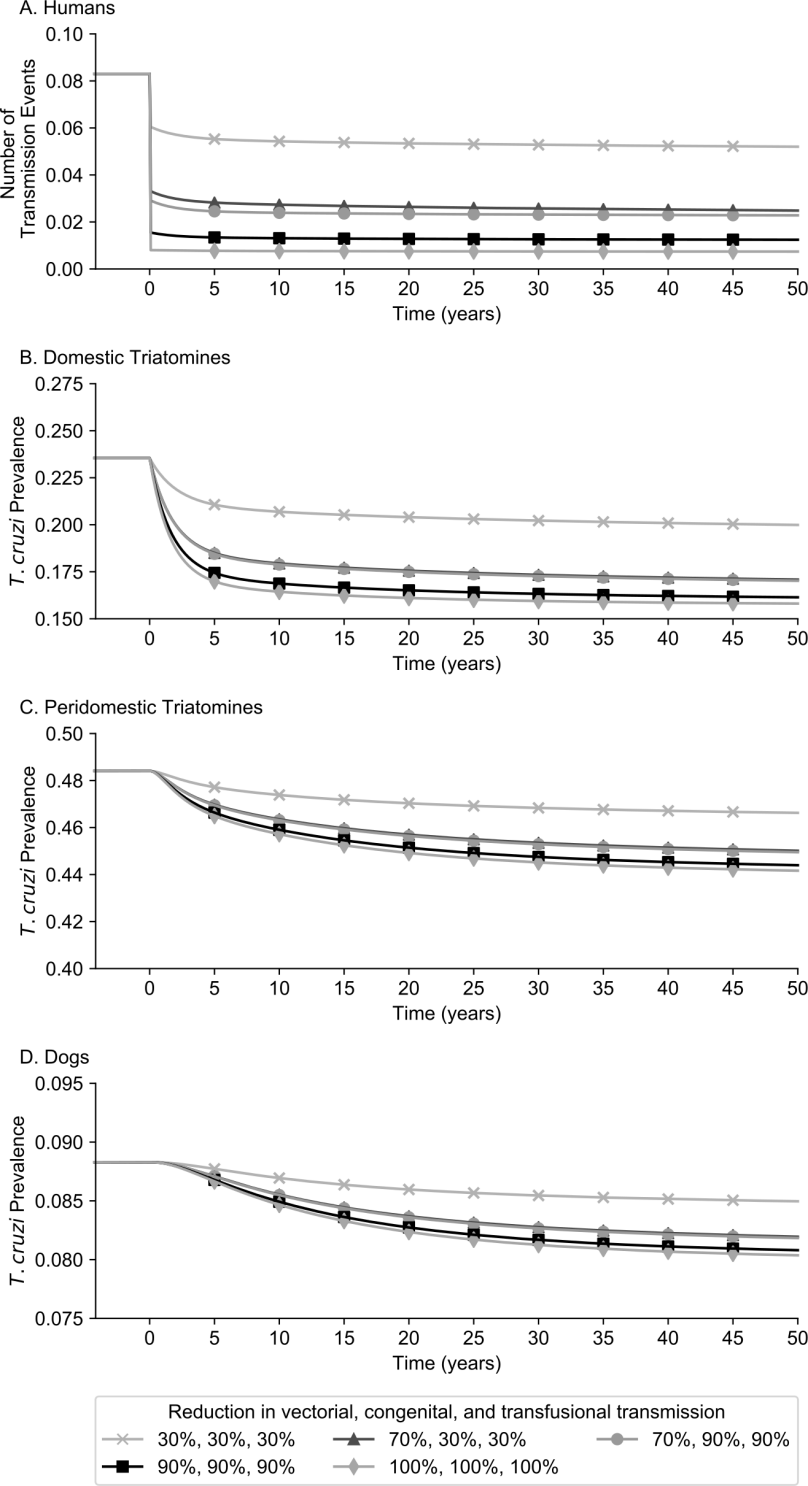
Impact of the degree of reduction of vectorial, congenital, and transfusional transmission on *T*. *cruzi* transmission events and seroprevalence over time in A) humans, B) domestic triatomines, C) peridomestic triatomines, and D) dogs.

Fig 4 and Table 3 show the impact of reducing all three transmission routes to varying degrees. After five years, there are two to five fewer Chagas cases per 2,000 persons, varying with the degree of interruption (Table 3); however, differences increase over time, with 25 fewer cases given 100% interruption of all three transmission routes. Stopping all three forms of transmission resulted in 0.2 transmission events over the first year and 0.5 over five years (compared to 1.0 and 5.0 with no interruption over one and five years, respectively); interrupting all forms by 30% resulted in 3.4 total events over five years. This corresponds to a 32% to 90% relative reduction (30% to 100% interruption in all forms) in new acute cases over five years. Interrupting all three transmission routes by 100% resulted in a human prevalence of 0.6% after 50 years. Transmission from vector to dog (ranging from 0.006 to 0.008 transmission events per month), followed by triatomine movements between habitats, and transmission from vector to human had the largest impact on the number of new transmission events (Fig 3C). Again, varying the three parameters most impactful with no transmission resulted in a range of 0.06 to 0.10 new acute cases per year (compared to 0.08 per year when held at middle values). Relative reductions in domestic triatomine prevalence over five years ranged from 10.5% to 27.8% (30% reduction in all types to 100% reduction in all types).

**Table 3:** Impact of interruption of domestic vectorial, congenital, and transfusional transmission to varying degrees on the number of humans in each Chagas disease state at given time points over the 50-year period in Yucatan, Mexico.

	Reduction in Vectorial, Congenital, and Transfusional Transmission, respectively					
	0%, 0%, 0%	30%, 30%, 30%	70%, 30%, 30%	70%, 90%, 90%	90%, 90%, 90%	100%, 100%, 100%
Year 0						
Acute	1.51	1.51	1.51	1.51	1.51	1.51
Indeterminate	30.56	30.56	30.56	30.56	30.56	30.56
Chronic	4.63	4.63	4.63	4.63	4.63	4.63
Year 5						
Acute	1.51	1.03	0.56	0.50	0.30	0.19
Indeterminate	30.56	29.7	28.79	28.66	28.25	28.04
Chronic	4.63	4.61	4.59	4.58	4.57	4.57
Year 15						
Acute	1.51	0.98	0.49	0.43	0.2	0.14
Indeterminate	30.56	27.39	24.31	23.90	22.62	21.97
Chronic	4.63	4.41	4.20	4.17	4.08	4.03
Year 25						
Acute	1.51	0.96	0.47	0.42	0.23	0.14
Indeterminate	30.56	25.52	20.78	20.20	18.29	17.32
Chronic	4.63	4.15	3.69	3.63	3.44	3.34
Year 35						
Acute	1.51	0.9	0.46	0.42	0.23	0.13
Indeterminate	30.56	24.06	18.05	17.37	14.99	13.79
Chronic	4.63	3.89	3.21	3.12	2.85	2.71
Year 45						
Acute	1.51	0.95	0.45	0.41	0.23	0.13
Indeterminate	30.56	22.9	15.93	15.21	12.47	11.11
Chronic	4.63	3.68	2.80	2.70	2.35	2.18

The differences between scenarios show the gains that can be achieved by increasing the degree of vector-host interruption at different points in time. Greater achievements could be made by increasing vector-host interruption alone than by increasing control of congenital and transfusional together (i.e., greater gains increasing vectorial from 30% to 70% than increasing congenital and transfusional from 30% to 90%). For example, if congenital and transfusional transmission were interrupted by 90%, further reducing vector-host contact from 70% to 90% in year two for two years would result in 1.0 total transmission event vs. 1.3 per 2,000 persons.

## Discussion

Our results show that even if the 2020 goals of interruption of domestic vectorial, congenital, and transfusional transmission in the Yucatan (and in other regions with similar epidemiological conditions) were achieved at 100% for 50 years, Chagas disease would still persist. While interrupting domestic vector transmission resulted in the greatest gains (up to an 83% relative reduction in new acute cases over five years), reduction of all three forms can achieve a slightly greater impact. Additionally, interrupting domestic transmission may lower the prevalence of *T*. *cruzi* in both domestic and peridomestic triatomines, however, it alone does not completely control Chagas disease in these populations after several years. In our model, meeting part of the 2020 goals does not completely control Chagas disease given that transmission in peridomestic settings is maintained and bugs are allowed to move between habitats. However, it should be noted both settings are closely linked, and while we only model reductions in the domestic setting (according to the 2020 goals), it is logistically feasible that vector control interventions like spraying could be applied in both settings. Housing improvements may help reduce the movement between the peridomestic and domestic habitats. Thus, interruption in both settings may be interrelated.

Our results also show that changes in human prevalence take years to manifest. Thus, complementary control approaches are needed to target the large population of already infected individuals. While indeterminate and chronic Chagas disease would still persist, the incidence of new cases of *T*. *cruzi* infection would be markedly reduced, as up to 82% of the reductions in transmission are achieved within one year. Thus, there is still enough of a time window to achieve substantial gains in those regions that have not yet started, or are early on, in working toward the 2020 goals. However, we model robust interruptions. In reality, this may be hindered by imperfect implementation, efficacy, and non-perfect compliance, which would take more time for impacts in the disease burden to fully manifest. Nonetheless, our results also show an important impact even if we fall short of the 2020 goals. For example, if only a 50% interruption is achieved in domestic vector-host contacts, we can see what was missed from not having achieved 100% interruption (46.1% vs. 83.0% relative reduction in the number of human transmission events over five years). By missing all three of these goals, there could be 0.1 to 1.8 more transmission events per 2,000 persons in five years (i.e., 5 to 90 per 100,000 persons). These represent missed opportunities and show what could have been gained by further reducing the interruption of one or more types of transmission.

If the 2020 goals in the Yucatan are achieved, although achieving a 90% relative reduction in transmission events, there would still be 25 new acute cases per 100,000 persons over five years. Given its long disease course, changes in prevalence take longer to manifest (prevalence of 1.6% at 5 years and 0.6% at 50 years). Thus, other complementary prevention and control measures would be needed to achieve a faster reduction in *T*. *cruzi* prevalence. For example, vaccines. Several Chagas vaccines are under development and in pre-clinical trials.[53] Both preventative and therapeutic vaccines would be cost-effective under a wide ranges of circumstances and scenarios.[54, 55] A preventative vaccine would be very cost-effective and even economically dominant (i.e., saving costs and providing health benefits) with a risk of infection as low as 1% and vaccine efficacy as low as 25%.[54] As for a therapeutic vaccine, even one meeting only the minimally acceptable target criteria ($200, two-dose, 80% efficacious) would be economically dominant over no vaccination and could provide substantial return on investment.[55] Other such measures include new medications, improved treatment seeking behavior, and other prevention and control strategies, such housing improvements which may be key to domestic interruption. These strategies may be able to overcome the current limitations of vector control (e.g., efficacy and vector resistance to insecticides[13, 14, 56]), and limited screening and treatment of pregnant woman and infants, and poor compliance with blood supply screening[17]. Additionally, strategies such as vaccination or other prophylactic approaches may avert transmission events that occur in other settings (e.g., peridomestic, sylvatic) and may protect against other forms of transmission (e.g., transfusional and oral routes).

These other complementary approaches may help with other important issues regarding Chagas transmission. Reinfestation, especially in areas with sylvatic populations[27, 28, 56, 57], can thwart the interruption of domestic transmission of Chagas disease. Reinfestation requires repeated use of some vector control strategies and therefore, while some vector control programs are successful, they need continued political, financial, and personnel support and resources over time. Thus, sustainability and long-term use can be an issue. Our future work could evaluate the impact of waning support over time.

Our model was developed to answer policy related questions about control of *T*. *cruzi* transmission and the role of non-human hosts on a larger scale than previous models.[10] It aims to explore potential target populations for interventions and to focus more on relevant outcomes, rather than to evaluate, explore, and understand the dynamic relationships of *T*. *cruzi* transmission. Our model can also help guide research and adaptive management (i.e., experimental management strategies) for transmission reduction. Thus, it is different from previous models of *T*. *cruzi* transmission and control that only include the domestic setting.[7, 9, 11], are at the household level (i.e., one house or small population)[7, 11, 17, 58], include only one form of transmission[9, 11, 14, 58], or evaluate complex biological interactions and transmissions to evaluate non-policy related issues[6, 8, 14, 59, 60].

### Limitations

All models are simplifications of real life and as such cannot represent every possible event or outcome. Our current model is deterministic in nature and does not include the full heterogeneity possible for Chagas disease transitions between states. Our model inputs were fit to disparate data of varying quality yet can be refined as new data become available. As Chagas disease is underdiagnosed and underreported, our estimates for *T*. *cruzi* seroprevalence in the absence of control measures are subject to limitations; however, we used the best available data for these parameters. We assumed a robust interruption in transmission that did not wane over time and assumed a constant reduction. While our model allows for differential infectiousness of humans in the three disease states, we assumed the same value for both indeterminate and chronic patients, as evidence suggests these patients have comparable levels of low parasitemia.[61] We also did not consider oral Chagas disease transmission nor account for seasonal effects. For simplicity, our model also does not include other biological states, transmission types, and outcomes for dogs nor other synanthropic wildlife (however, dogs and chickens serve as reservoir and dead-end populations, respectively). Given lack of data on transmission rates for parameterization, we did not further stratify the infection state in dogs to include acute and chronic disease, nor did we consider oral transmission. Likewise, we did not include the impact of predation rate on vectors by dogs, given we modeled a stable bug population. While there is a possibility that excluding these factors may affect results, most likely they would not as we calibrate the simulation to a certain prevalence in dogs and this would be maintained regardless of the number of dog disease states and transmission types. This prevalence is maintained by the vector to dog transmission rate, thus highlighting its importance to our model; however, we note that our resulting calibrated value for the infectiousness of dogs was lower than values reported in the literature.[62] Our future work can further incorporate these factors.

### Conclusions

Our results suggest that achieving the 2020 Sustainable Development Goals of 100% interruption and control of domestic sectorial, congenital, and transfusional transmission in the Yucatan and other regions with similar epidemiological conditions fall short of completely interrupting *T*. *cruzi* transmission, despite considerably reducing the number of new Chagas cases. Thus, complementary approaches and other prevention and control measures (e.g., peridomestic vector control, vaccines and increased healthcare utilization) are needed to fully interrupt Chagas disease transmission. Even if these goals are missed, most gains are achieved within the first year of implementation, thus the goals should be actively pursued.

## Supporting information

S1 Text Technical appendix. (DOCX)
