# Supplementary material for: Are the London Declaration’s 2020 goals sufficient to control Chagas disease?: Modeling scenarios for the Yucatan Peninsula
Source: PLoS Negl Trop Dis. 2018 Mar 19;12(3):e0006337. doi: 10.1371/journal.pntd.0006337 (PMC5875875; doi:10.1371/journal.pntd.0006337)

**S1 Text.**

*Habitats*

Our model includes two habitats (*i*), the domestic (*i=1)* and peridomestic (*i=2*) habitats. All populations can move between these two habitats with the exception of chickens, which remain in the peridomestic habitat. *T. cruzi* transmission occurs in both habitats, further described below.

*Triatomine Bugs/Vectors*

Triatomine bugs can be susceptible (*S*, not infected with *T. cruzi* and able to become infected) or infectious (*I*, infected with *T. cruzi* and able to transmit to others upon biting, i.e., vectors). The following formulas describe the disease states for bugs:

$$\frac{dS_{V1}}{dt}=b_{V1}-\gamma_{V1}S_{V1}-d_{V}{S_{V1}+ \rho S}_{V2}- {\omega S}_{V1}$$

$$\frac{dI_{V1}}{dt}=\gamma_{V1}S_{V1}-d_{V}I_{V1}+{\rho I}_{V2}-{\omega I}_{V1}$$

$$\frac{dS_{V2}}{dt}=b_{V2}-\gamma_{V2}S_{V2}-d_{V}{S_{V2}+ \omega S}_{V1}- {\rho S}_{V2}$$

$$\frac{dI_{V2}}{dt}=\gamma_{V2}S_{V2}-d_{V}I_{V2}+{\omega I}_{V1}-{\rho I}_{V2}$$

where 1 and 2 represent, domestic and peridomestic, respectively. Additionally, *b_v_* is the number of bug births, *d_v_* is the triatomine death rate, *ρ* is the rate at which bugs in the peridomestic habitat move to the domestic habitat, *ω* is the rate at which bugs in the domestic habitat move to the peridomestic habitat. The number of bug births is determined by the birth rate, carrying capacity and total number of triatomines in each setting by the following formula:

$$b_{Vi}=birthrate*N_{Vi}*\frac{carryin{g capacity}_{i}-N_{Vi}}{carryin{g capacity}_{i}}$$

The following formulas determine the force of infection in domestic (*ϒ_V1_*) and peridomestic (*ϒ_V2_*) settings:

$$\gamma_{V1}=\beta\left[ \frac{{p_{D}f_{D}\theta}_{D}I_{D}+p_{H}\left( {f_{H}((\theta}_{A}A_{H}+\theta_{I}{(I}_{H}+\theta_{C}) \right)}{p_{H}{f_{H}N}_{H1}+p_{D}{f_{D}N}_{D}} \right](1-\pi_{id})$$

$$\gamma_{V2}=\beta\left[ \frac{{p_{D}(1-f_{D})\theta}_{D}I_{D}+p_{H}\left( {(1-f}_{H})((\theta_{A}A_{H}+\theta_{I}(I_{H}+C_{H}) \right)}{p_{H}{{(1-f}_{H})N}_{H}+p_{D}{(1-f_{D})N}_{D}+p_{C}N_{C}} \right]$$

where *β* represents the triatomine biting rate, *Θ* is the probability of transmission (or infectivity), *N_H_*, *N_D_*, and *N_C_* are the number in the human, dog, and chicken populations, respectively; *p_H_*, *p_D_*, and *p_C_* describe the vector feeding proportions for humans, dogs, and chickens, respectively, *f_H_* and *f_D_* represent the proportion of time spent by humans and dogs, respectively, in the domestic setting, and $\pi_{id}$ is the effectiveness of intradomiciliary interruption.

*Humans*

Each member of the human population falls into one of four mutually exclusive states: susceptible (*S_H_*, not infected with *T. cruzi* and able to become infected), acute stage Chagas disease (*A_H_*, infected with *T. cruzi* and able to transit, exhibit mild and nonspecific symptoms, but in some cases can show specific symptoms such as Romaña’s sign or can be serious and life-threatening, and person has microscopically detectable parasitemia), indeterminate stage Chagas disease (*I_H_*, asymptomatically infected with *T. cruzi* and able to transmit), and chronic stage Chagas disease (*C_H_*, infected with *T. cruzi*, able to transmit, and show symptoms of chronic disease such as cardiomyopathy and/or megaviscera). These states and the transmission between them are described by the following four equations:

$$\frac{dS_{H}}{dt}=\left( 1- \right.\gamma_{c})b_{H}-\gamma_{H}S_{H} {- \gamma}_{t}S_{H}-d_{H}S_{H}$$

$$\frac{dA_{H}}{dt}={\gamma_{c}b_{H}+\gamma}_{H}S_{H}+\gamma_{t}S_{H}-\alpha_{H}A_{H}-\left( d_{H}+\mu_{A_{H}} \right)A_{H}$$

$$\frac{dI_{H}}{dt}=\alpha_{H}A_{H}-{\lambda_{H}I}_{H}-d_{H}I_{H}$$

$$\frac{dC_{H}}{dt}={\lambda_{H}I}_{H}-(d_{H}+\mu_{C_{H}})C_{H}$$

where *b_H_* is the number of human births, *d_H_* is the human death rate from natural/all causes, $\mu_{A_{H}}$ is the probability of Chagas related mortality in the acute phase of disease, and $\mu_{C_{H}}$ is the probability of Chagas related mortality in the chronic phase. Two variables, $\alpha$*_H_ and λ_H_*, describe the rate of movement from the acute phase to the indeterminate phase and the indeterminate phase to the chronic phase, respectively.

$\gamma_{c}$ is the force of infection in humans from congenital transmission, and is defined by the following equation:

$$\gamma_{c}=\left[ \varphi_{wr}(1-\pi_{c})\epsilon_{c}\frac{A_{H}+I_{H}+C_{H}}{N_{H}} \right]$$

where $\varphi_{wr}$ is the relative prevalence of Chagas among women of reproductive age compared to the rest of the population, $\pi_{c}$ is the effectiveness of congenital interruption and $\epsilon_{c}$ is the probability of congenital transmission.

*ϒ_H_* is the force of infection in humans from vectors, and is defined by the following equation:

$$\gamma_{H}=\left[ (1-\pi_{id})f_{H}\frac{\epsilon I_{V1}}{N_{H}}\left( \beta\frac{p_{H}}{p_{D}+p_{H}} \right)+(1-f_{H}) \frac{\epsilon I_{V2}}{N_{H}}\left( \beta\frac{p_{H}}{p_{D}+p_{H}+p_{C}} \right) \right]$$

where $\pi_{id}$ is the effectiveness of interdomiciliary interruption, *β* is the vector biting rate, *ε* is the probability of *T. cruzi* transmission to humans given the bite of an infected vector, *f_H_* is the proportion of time humans spend in domestic settings, and *p_H_*, *p_D_*, and *p_C_* are the vector feeding proportions for humans, dogs, and chickens, respectively.

$\gamma_{t}$is the force of infection in humans from transfusion/organ transplantation, and is defined by the following equation:

$$\gamma_{t}=\left[ p_{t}(1-\pi_{t})\epsilon_{t}\frac{A_{H}+I_{H}+C_{H}}{N_{H}} \right]$$

where $p_{t}$ is the rate at which a random individual in the population will receive a blood transfusion or an organ transplant (probability of receiving blood transfusion or organ transplant during a month), $\pi_{t}$ is the efficacy of blood/organ donation screening for Chagas infection, and $\epsilon_{t}$ the probability that receiving infected blood/organ results in infection of the receiver.

It should be noted that both the force of infection in humans from congenital transmission and from transfusion/organ transplantation are mechanistic, as both are dependent on human prevalence of *T. cruzi*, which is dependent on vectorial transmission. Additionally, π represents any number of measures that interrupt transmission (e.g., housing improvements, bed nets, indoor residual spraying, screening, treatment, etc.) as it reduces the force of infection. While some interventions may reduce the number of bugs (i.e., indoor residual spraying), this effectively reduces the contact rate/force of infection; thus, is modeled generically to represent a number of interventions or the use of them in combination.

*Non-human Hosts: Dogs*

Members of the dog population can be either susceptible (*S_D_*) or infectious (*I_D_*), with movement between these two compartments described by the following force of infection (*ϒ_D_*) equation.

$$\frac{dS_{D}}{dt}=b_{D}N_{D}-\gamma_{D}S_{D}-d_{D}S_{D}$$

$$\frac{dI_{D}}{dt}=\gamma_{D}S_{D}-d_{D}I_{D}$$

$$\gamma_{D}=\left[ \tau_{D}\frac{\epsilon I_{V1}}{N_{D}}\left( \beta\frac{p_{D}}{p_{D}+p_{H}} \right)+(1-\tau_{D}) \frac{\epsilon I_{V2}}{N_{D}}\left( \beta\frac{p_{D}}{p_{D}+p_{H}+p_{C}} \right) \right]$$

Here, *b_D_* and *d_D_* are birth and death rates of dogs, respectively. *ε* is the probability of *T. cruzi* transmission to dogs given the bite of an infected vector. As already described, *f_D_* represents the proportion of time spent by dogs in the domestic setting, *β* is the vector biting rate, and *p_H_*, *p_D_*, and *p_C_* are vector preferences for humans, dogs, and chickens, respectively.

*Dead-End Hosts: Chickens*

Chickens serve as dead end hosts (i.e., able to become infected but unable to transmit back to triatomine species). In our model, chickens are an alternate food source for triatomines, and are incorporated into the force of infection formulas above by accounting for triatomine feeding preference for them.

*Model Calibration and Equilibrium Conditions*

Our model was calibrated to *T. cruzi* prevalence in triatomines and *T. cruzi* seroprevalence in humans and in dogs in the Yucatan, Mexico. Because transmission parameters (i.e., to and from vectors to hosts, from mother to child, and via blood transfusion) and triatomine feeding preferences are highly variable and/or not well defined in the literature, these values were used as the search space during the calibration process. Two methods of calibration were performed to identify an acceptable range of values for the calibrated parameters. To find the set of parameter values that produced prevalence values closest to our baseline scenario assumptions, we used a genetic algorithm to search our twelve-dimensional parameter space. At the beginning of each step in the genetic algorithm, a population of parameter value combinations was created and used to run our model. The fitness of each member of the population was determined by a weighted mean squared error between the assumed prevalences and the simulated prevalences. We weighted human prevalence more highly in the error computation to ensure the modeled prevalence matched most closely to the epidemiologic data. The ratio of weights for human, vector, and dog prevalence, based on their contribution to the mean squared error, was about 14.5:5:1, respectively. At the end of each step, the members of the population with the highest fitness were combined and reproduced to generate the next generation of the population, and random mutations were introduced to prevent the population from getting stuck in local minima. This process was repeated until the majority of the population had converged to a common set of parameter values. This method found a set of parameter values that produced prevalence values within 0.5% and 10% error of the assumed prevalence for humans and vectors, respectively (Table A). The dog prevalence was found within 34% error, as it carried the smallest weight. This resulted in a total percent error of 43.8%.

Table A.

|  | **Human** | **Vector** | **Dog** |
| --- | --- | --- | --- |
| Target values | 0.01850 | 0.3250 | 0.14580 |
| Calibration method 1 | 0.018412 | 0.3554 | 0.09627 |
| Percent error | 0.47% | 9.34% | 33.97% |

With this parameter set as a guide, the second calibration method searched for sets of parameter values that generated prevalence values with between 45% and 51% total percent error of the assumed baseline prevalences. These sets reflected the uncertainty around the assumed baseline prevalences and model’s input parameter values. This method divided each of the twelve calibration parameters into three intervals and performed a local optimization of the previously described error function within each of the combinations of intervals.

We ran a 500-year burn-in period; at 250 years, *T. cruzi* prevalence in humans (0.018354) was within .05% of the calibrated value). To test the stability of our model, we checked to see if our model returned to equilibrium after perturbing the system. After the burn-in period, we applied 100% interruption of all three forms of transmission for 50 years, after which we turned off the interruption and ran the model for another 450 years. These Figures (FigA-C) show the resulting prevalence in each population and return to equilibrium.

Fig A.


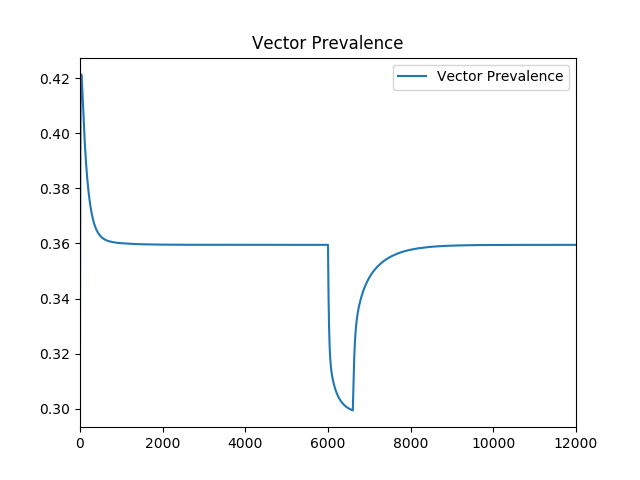


Fig B.


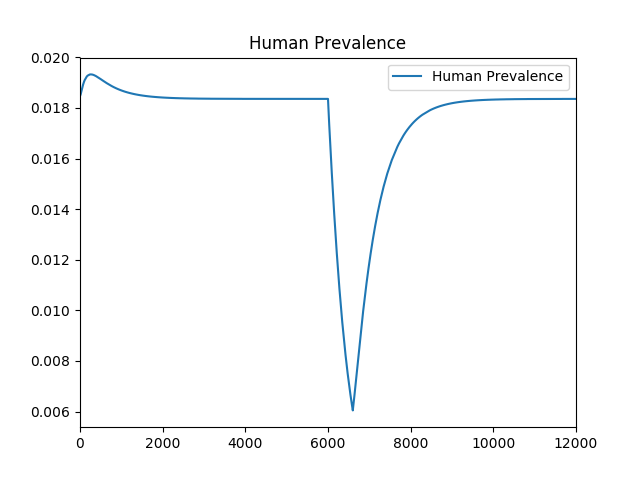


Fig C.


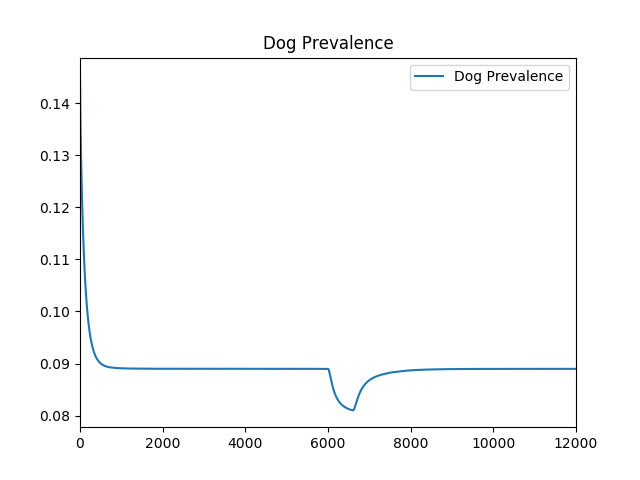

Supplement: S1 Text — (DOCX) [file pntd.0006337.s001.docx]
